# Supplementary material for: Identifying Soybean Pod Borer (Leguminivora glycinivorella) Resistance QTLs and the Mechanism of Induced Defense Using Linkage Mapping and RNA-Seq Analysis
Source: Int J Mol Sci. 2022 Sep 18;23(18):10910. doi: 10.3390/ijms231810910 (PMC9504297; doi:10.3390/ijms231810910)
Supplement: Supplementary file 1 [file ijms-23-10910-s001.zip › Illustrated with Figure S1-S7.pdf]

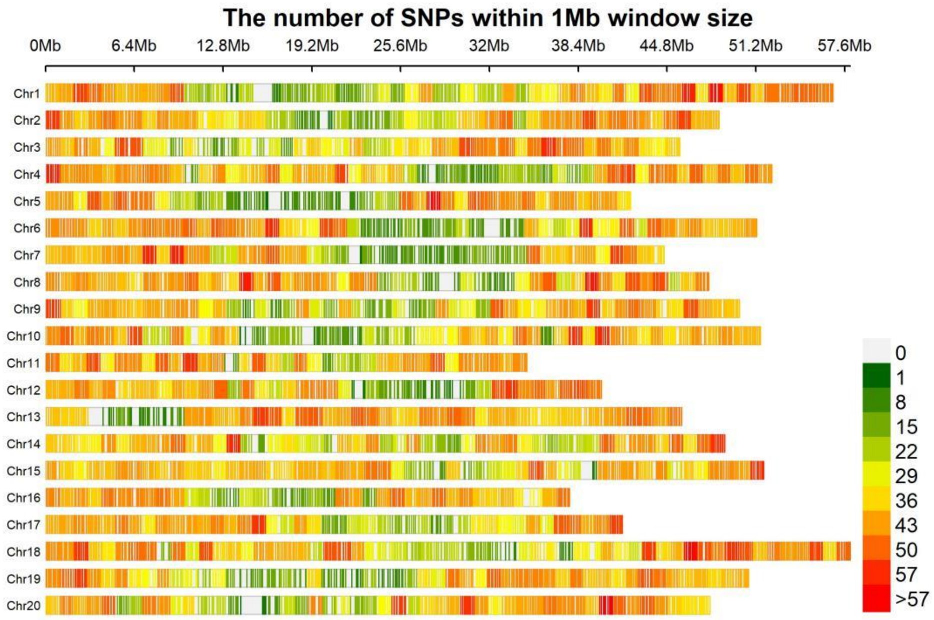

Figure S1. Raw SNPs of F_2 populations based on GTBS method. The ruler on upper side indicated the length of chromosomes using cM (centimorgan) as the unit. The color bar represents the number of SNPs in the 1Mb window, the more red the color the more SNPs.

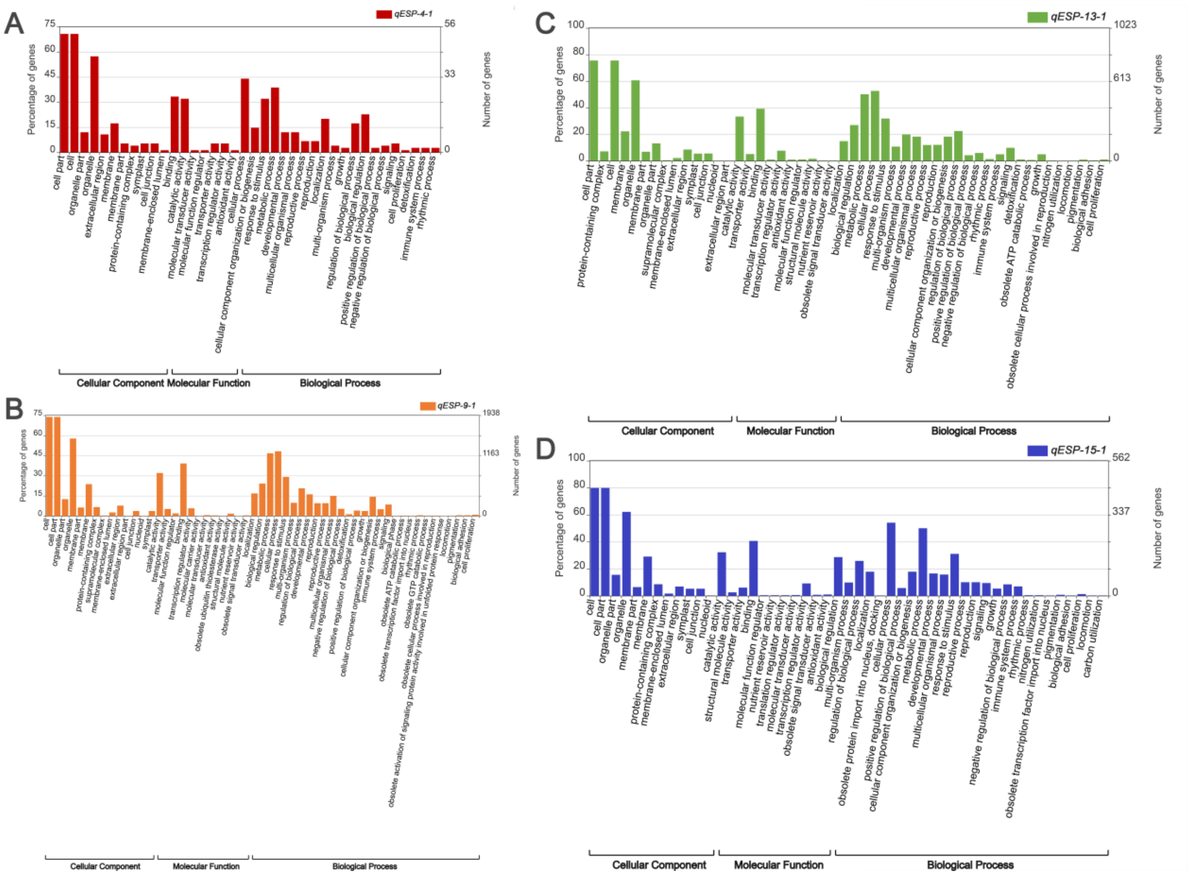

Figure S2. GO term analysis of interval genes in four ESP-related QTLs using WEGO 2.0. (A) The interval genes of *qESP-4-1*. (B) The interval genes of *qESP-9-1*. (C)The interval genes of *qESP-13-1*. (D)The interval genes of *qESP-15-1*. The ruler on the left side indicates the number of genes contained in the entry as a percentage of the total number of genes, and the ruler on the right indicates the number of genes contained in the term.

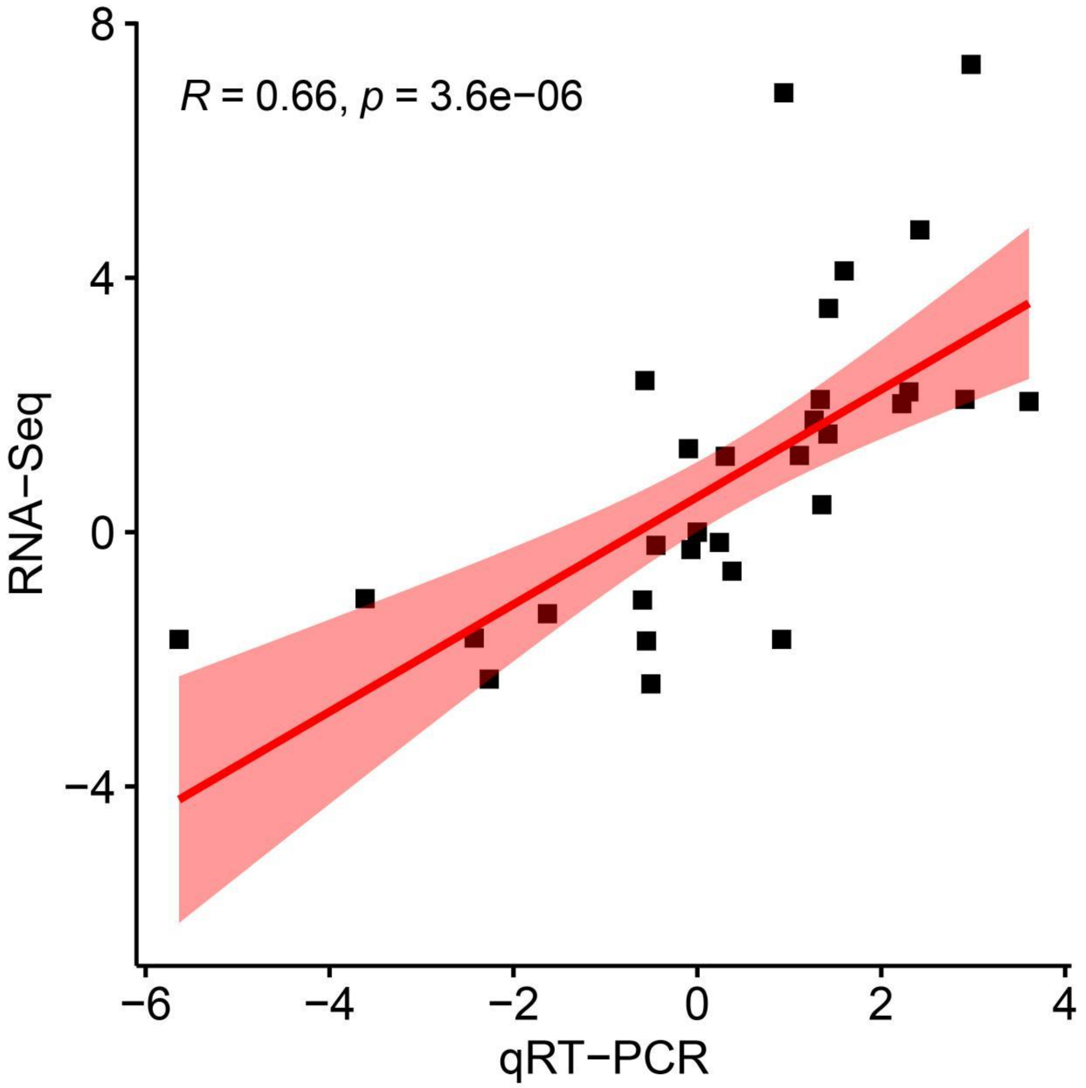

Figure S3. Comparison of relative expression measured by qRT-PCR and RNA-Seq (Log2FC). The x-axis is the result of qRT-PCR, and the relative expression is converted using the $2^{-\Delta\Delta_{Ct}}$ method and plotted as log2($2^{-\Delta\Delta_{Ct}}$). The y-axis is the result of RNA-Seq, and the expression is normalized to FPKM and converted to log2(Fold Change) with reference to qRT-PCR.

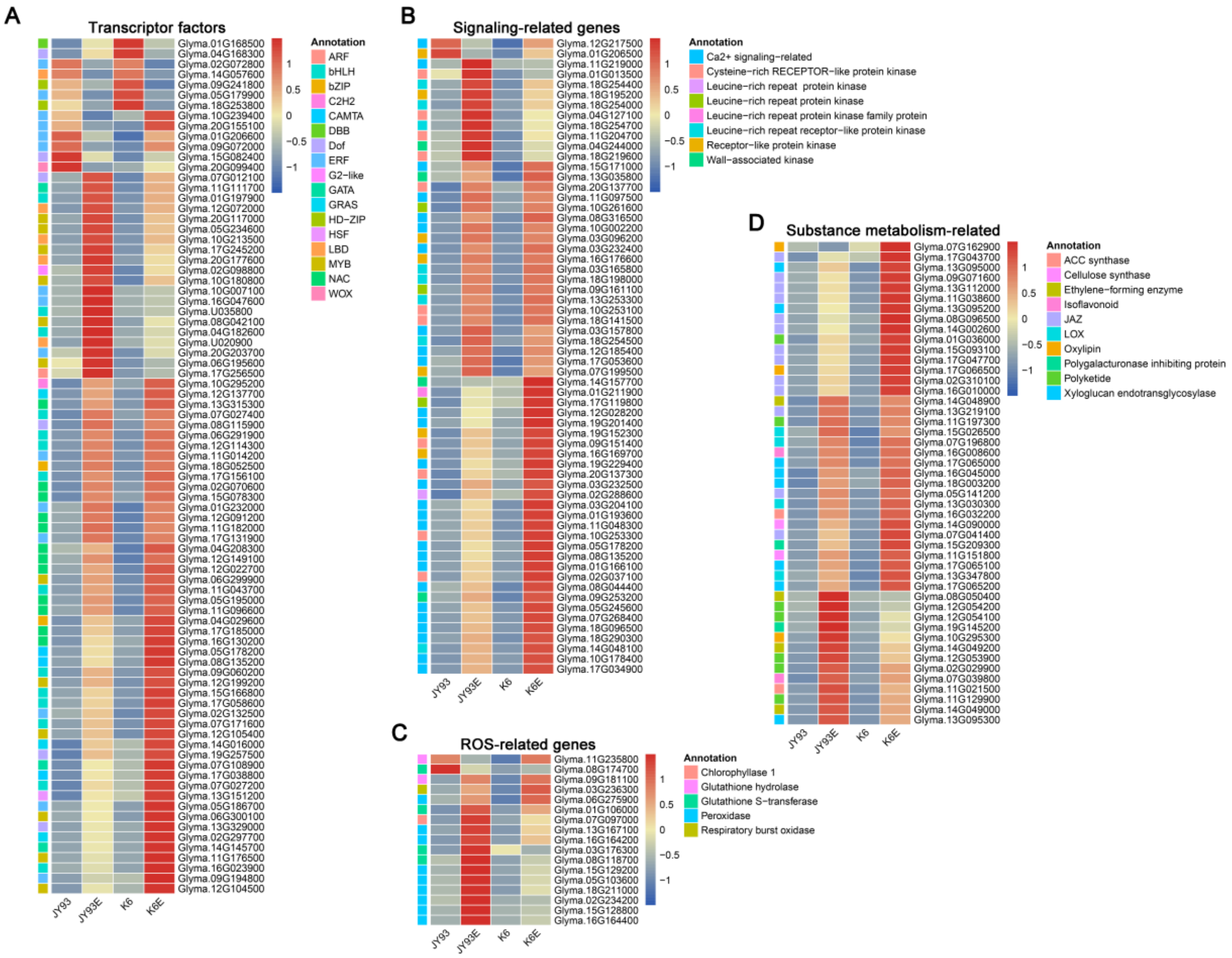

Figure S4. Expression levels of CRGs of interest. (A) TFs. (B) The genes associated with signal transduction pathway. (C) ROS-related enzymes. (D) Genes related to hormones, secondary metabolites and cell wall substances. The color bar on right side indicated the expression levels of certain genes by Z-score method, red is high expression, blue is low expression.

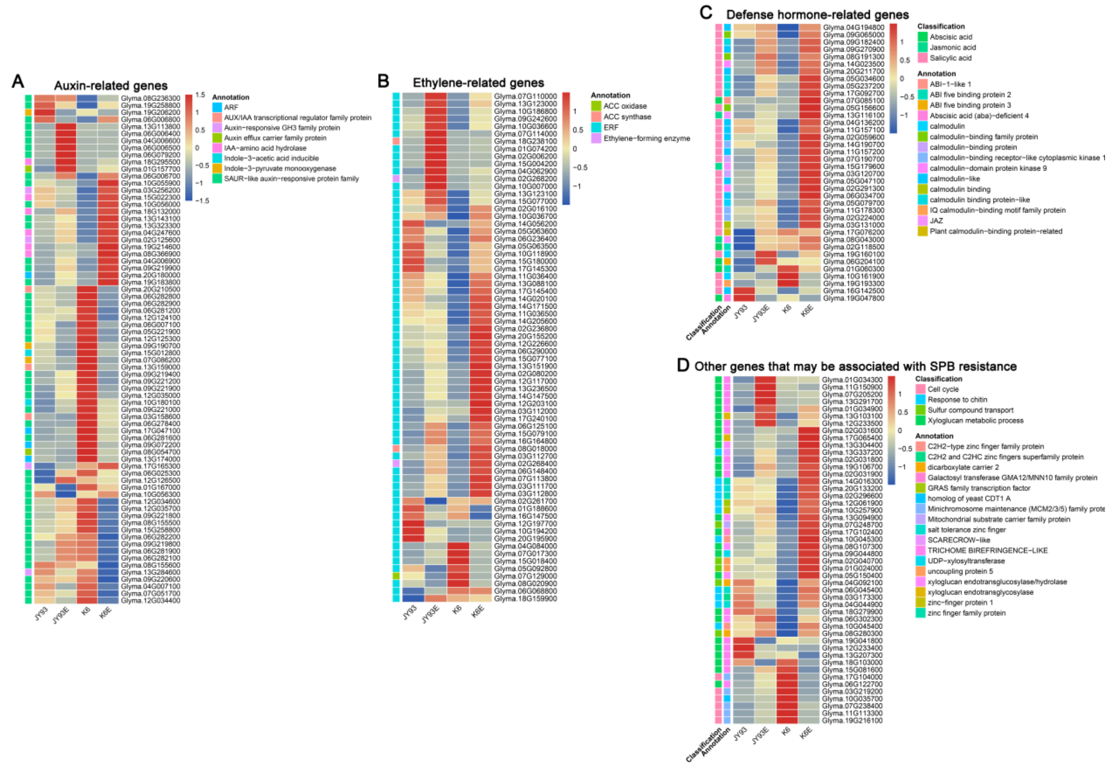

Figure S5. Expression levels of DRGs of interest. (A) Auxin-related genes. (B) Ethylene-related genes. (C) Defense hormone-related genes. (D) Other genes that may be associated with SPB resistance. The color bar on right side indicated the expression levels of certain genes by Z-score method, red is high expression, blue is low expression.

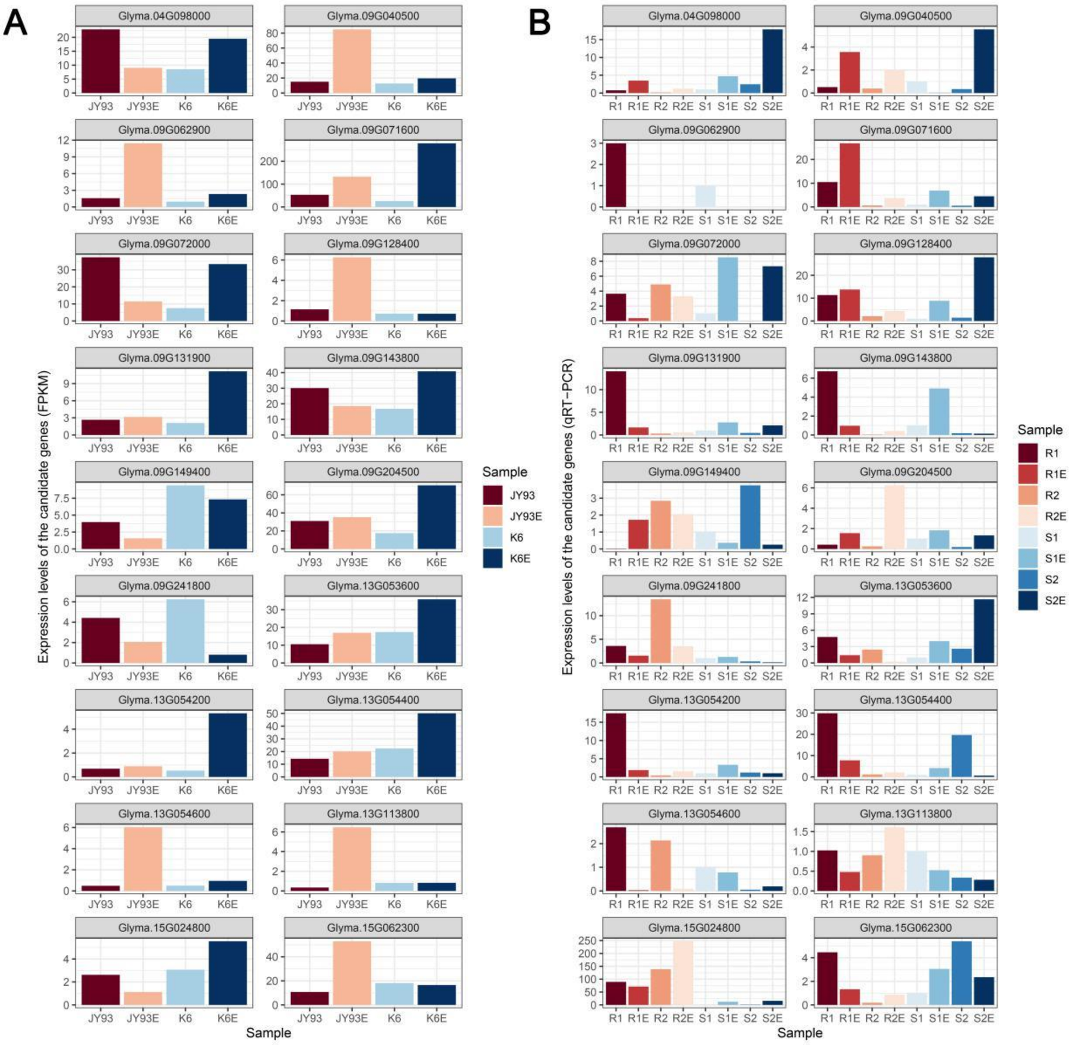

Figure S6. Expression levels of the initial selected candidate genes.(A) RNA-Seq data of the candidate genes in the mapping parents. The scale on the left indicates the expression levels (FPKM). (B) qRT-PCR data of the candidate genes in the four soybean varieties. qRT-PCR data of the candidate genes in the four soybean varieties. Among these, two of which were resistant varieties [R1 (ESP=2.48%, n=15), R2(ESP=2.49%, n=15)] and the other two were

susceptible varieties [S1(ESP=16. 00%, n=15), S2 (ESP=17.59%, n=15)]. The scale on the left indicates the relative expression levels ($2^{-\Delta\Delta Ct}$).

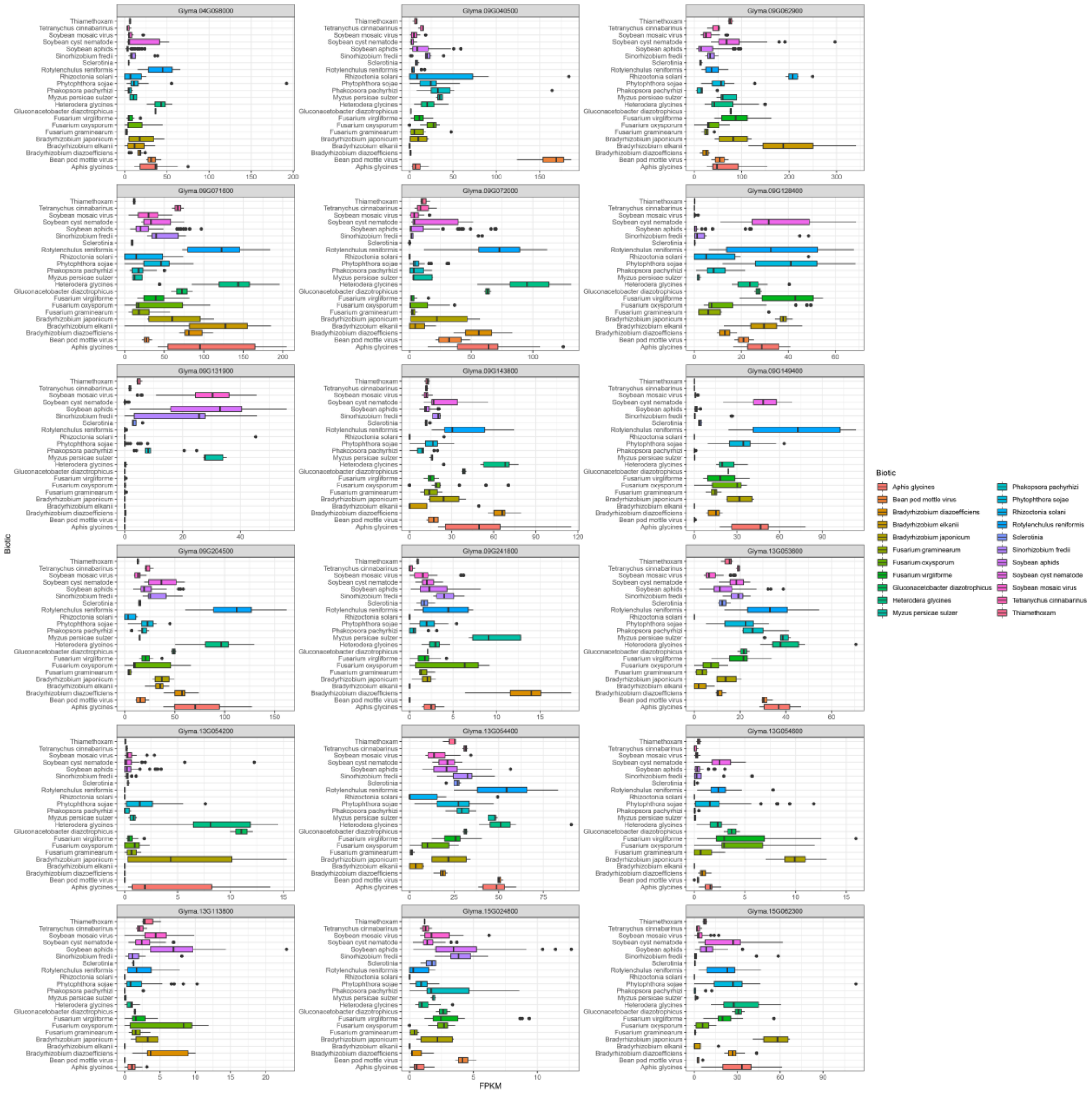

Figure S7. Expression profiles of candidate genes for other biotic stresses from public data (http://ipf.sustech.edu.cn/pub/soybean/). The x-axis is the expression levels of candidate genes in other biotic stresses (FPKM), the y-axis is the biotic stresses name.
